# Delivery after an obstetric anal sphincter tear

**DOI:** 10.1007/s00404-020-05550-1

**Published:** 2020-04-23

**Authors:** J. Pirhonen, K. Haadem, M. Gissler

**Affiliations:** 1 The Norwegian Continence and Pelvic Floor Center, University Hospital of North Norway, P.O. Box 96, 9038 Tromsø, Norway; 2 Department of Obstetrics and Gynecology, Helsingborg Hospital, Lund’s University, Helsingborg, Sweden; 3 Information Services Department, Finnish Institute for Health and Welfare, Helsinki, Finland; 4 Department of Neurobiology, Care Sciences and Society, Karolinska Institute, Stockholm, Sweden

**Keywords:** Obstetric anal sphincter injuries, Subsequent delivery, Scandinavian countries

## Abstract

**Purpose:**

The present study aimed to assess the risk of obstetric anal sphincter injuries (OASIS) of a subsequent delivery after the previous OASIS in countries with low (Finland) and high rates (Norway and Sweden) of OASIS.

**Methods:**

This population-based case–control study included women who experienced OASIS 1997–2002. 26,598 women with OASIS were included from countries with low (Finland) and high (Norway and Sweden) OASIS incidences. Each case was matched with one background-adjusted control without OASIS. A follow-up data, including all subsequent deliveries between 1998 and 2011 were then collected. Statistics significances were calculated using chi-square test, test for relative proportions and Students *t* test, where appropriate.

**Results:**

OASIS in the first birth was associated with increased recurrences in subsequent births, 6.9% vs. 1.7% in Norway (*p* < 0.001); 4.5% vs. 0.7 (*p* < 0.001) in Sweden; and 2.1% vs. 0.8% in Finland (*p* = 0.038). In Norway, more than two deliveries occurred in 4.8% of cases and 6.2% of controls (*p* = 0.001), 4.2% vs. 5.1% in Sweden (*p* < 0.001), and 5.7% vs. 6.3% in Finland (*p* = 0.572). For women with OASIS in a previous delivery, the rates of cesarean deliveries in subsequent pregnancies were 16.4% (7.9% for controls) in Norway, and 16.3% (6.0% for controls) in Sweden, and 50.2% (14.2% for controls) in Finland. In all countries, the differences between cases and controls were significant (*p* < 0.001).

**Conclusion:**

Next deliveries after OASIS are associated with increased frequency of new OASIS, more cesarean deliveries, and less subsequent deliveries in the high-risk population than women without previous OASIS.

## Introduction

A common outcome of vaginal delivery is perineal trauma. The most serious among these injuries are third- and fourth-degree lacerations or obstetric anal sphincter injuries (OASIS). Mechanical damage of the anal sphincter muscles is assumed to be the most important risk factor for anal incontinence and anorectal symptoms in otherwise healthy women, thus, this condition can significantly reduce the quality of life [1–4]. Studies have reported that 30–50% of women with OASIS experience anal incontinence during their lifetime [1, 5]. Symptoms of anal incontinence may affect women’s social, psychological, and sexual life [6, 7]. Moreover, a previous OASIS can increase the risk of recurrence in subsequent deliveries. Therefore, it is important to prevent these injuries [8].

A better understanding of risks is necessary when counseling women with a prior OASIS. The risk of recurrence is a major factor in planning the mode of a subsequent birth. In some studies, women with prior OASIS were reported to be more likely to have a cesarean delivery for their next birth [9, 10].

Historically, the OASIS rate in Finland has been significantly lower than in other Scandinavian countries, e.g., 1% in Finland compared to 4% in Sweden and Norway [11].

Several hundreds of studies have investigated the risk factors of OASIS and the short-term complications that can occur after OASIS. However, some studies have investigated the obstetric consequences of OASIS in a subsequent pregnancy [12, 13]. The present study was designed primarily to assess the prevalence of OASIS in countries with low (Finland) and high rates (Norway and Sweden) of OASIS, and secondly to assess the prevalence of OASIS in subsequent pregnancy among women with a previous OASIS.

## Methods

We performed a population-based case–control study. Data were retrieved from the Medical Birth Registries (MBR) in Finland, Norway, and Sweden. The registry information was based on standardized forms completed by midwives in the delivery ward shortly after delivery. Only anonymized data were used. Thus, informed consent from participants was not required. For baseline data, all women who experienced OASIS in 1997–2002 were included. When possible, third- (external and/or internal sphincter damaged) and fourth-degree (external and internal sphincter and rectum’s mucous membrane damaged) perineal tears were identified from the ICD-10 codes, ‘O70.2’ and ‘O70.3’, respectively (it was not possible to differentiate between third- and fourth-degree tears in Norway or Sweden, and the presented data combined both third- and fourth grade of tears in Norway and Sweden). For cases, a matched control without OASIS was chosen and were matched for the following variables: (1) delivery year, (2) maternal age, (3) parity for vaginal deliveries, (4) an index delivery that was either a spontaneous vaginal delivery or an instrumental delivery, (5) birthweight either below or above 4000 g, (6) when multiparous, none had experienced an earlier anal sphincter tear and (7) gestational age above 22 weeks.

The follow-up included the subsequent deliveries after OASIS between 1998 and 2011. For the follow-up period, we collected the following information: (1) date of delivery (month/year), (2) method of delivery [a. spontaneous or instrumental (> 99% vacuum delivery), b. cesarean], (3) maternal age at delivery, (4) gestational age in weeks, (5) induction of labor, (6) epidural analgesia, (7) episiotomy, (8) presentation (just cephalic presentation), (9) birthweight, (10) Apgar score (at 5 min in Norway and Sweden, but at 1 min in Finland, because 5 min data were not collected in 1990–2003), and (11) number of subsequent children after OASIS.

The Finnish data was retrieved from the Finnish MBR with linkage to the Hospital Discharge Registry. Both registries were maintained by the Finnish Institute for Health and Welfare in Finland. Norwegian data was retrieved from The Norwegian MBR maintained by the Norwegian Institute of Public Health. Swedish data were retrieved from the Swedish MBR, maintained by the National Board of Health and Welfare. The study protocol was ethically approved in Finland by the Finnish Institute for Health and Welfare (granted 16 October 2008reference number 2777/605/2007). In Norway, approval was granted by the MBR (14-1753). In Sweden, the study was approved by the Ethics Committee of the University of Lund (D.nr 2014/747). Furthermore, the Finnish data were linked to data on induced abortions and sterilizations (Finnish Institute for Health and Welfare) and adoptions (Central Population Registry). This was not possible in Norway and Sweden.

Statistical significance was calculated by using Chi-square test, test for relative proportions and Students *t* test, where appropriate. All statistical analyses were performed with SAS 9.3 (SAS Institute Inc. 2011). *p* < 0.05 was considered statistically significant. The number of missing data varied between 0 and 1.0%.

## Results

The number of deliveries were equivalent between cases and controls in all countries. The total numbers were: 1001 deliveries in Finland, 10,327 in Norway, and 15,270 in Sweden (Table 1). Among the women who had experienced OASIS (cases) in Finland the injury occurred during the women’s first pregnancy for 72%, for 24% of women after two deliveries, and for 4% of women after three or more deliveries. In Norway and Sweden, new OASIS occurred for 74% and 69%, respectively, during the first delivery; 22% and 25%, respectively, during the second delivery, and 6% and 6%, respectively, during three or more deliveries.

**Table 1:** Basic characteristics of study participants data from three Nordic participating countries

Characteristic	Finland			Norway			Sweden		
	Cases	Controls	*p*	Cases	Controls	*p*	Cases	Controls	*p*
*N*	1001	1001		10,327	1037		15,270	15,270	
Epidural	56.7%	53.6%	0.164	28.2%	29.1%	0.148	42.6%	42.8%	0.793
Episiotomy	58.3%	67.4%	< 0.001	26.0%	25.0%	0.114	14.3%	15.5%	0.004
Normal Apgar (5 min)	93.0%*	94.2%*	0.273	98.4%	97.5%	0.011	98.6%	98.6%	0.8
Normal infant presentation^&^	93.6%	95.6%	0.048	92.9%	94.7%	< 0.001	7.3%	6.1%	< 0.001
Infant weight ≥ 4000 g^†^	30.4%	30.3%		33.8%	33.8%		33.5%	33.5%	
Cesarean delivery	11.2%	9.1%	0.120	8.2%	6.6%	< 0.001	7.3%	6.1%	< 0.001
Induction of labour	17.5%	15.5%	0.228	12.2%	12.3%	0.815	10.9%	9.8%	0.003

Cases had experienced an anal sphincter laceration; matched vs controls had no history of anal sphincter

*Finland: only 1-min Apgar scores were available for the study years

^†^Matching criteria

^&^Occiput anterior

The delivery characteristics are presented in Table 1. The frequency of epidural analgesia was not significantly different between cases and controls, but among the three countries, epidural analgesia occurred most frequently in Finland. The frequency of episiotomy was higher in controls than in cases in Finland and Sweden. However, there was a great difference in the use of episiotomy between these countries. Cases had better Apgar scores than controls in Norway, but no differences were observed in Finland or Sweden. A normal fetal presentation (occiput anterior) was found more often among controls than among cases, in Finland and Norway, but the opposite was true in Sweden. In all three countries, cases had more cesarean deliveries than controls. In Norway and Sweden, this difference was statistically significant. In Sweden, delivery was induced more frequently in cases than in controls (Table 1).

Gestational age for cases and controls was: (1) 22–36 weeks 3.3% vs. 2.6%, 37–41 weeks 94.2% vs. 92.0%, and at least 42 weeks 2.5% vs. 5.4% in Finland (*p* = 0.026); (2) 22–36 weeks 5.2% vs. 1.7%, 37–41 weeks 81.8% vs. 86.0%, and at least 42 weeks 12.9% vs 12.3% in Norway (*p* < 0.001); and (3) 22–36 weeks 1.7% vs. 5.4%, 37–41 weeks 90.1% vs. 86.2%, and at least 42 weeks 8.3 vs. 8.4% in Sweden (*p* < 0.001).

The births in subsequent pregnancies among women with and without OASIS during the first pregnancy are presented in Table 2. Both in Finland and in Norway, 64–65% of women delivered at least once after OASIS whereas the corresponding number for Sweden was 69.5% (*p* < 0.001 for both). However, there was no statistically significant difference between cases and controls in any of the three countries (matching criteria) (Table 2). The number of cesarean deliveries in subsequent deliveries was significantly higher among women with OASIS in their first delivery than among controls, in all three countries (Table 3). Similarly, the rates of elective cesarean deliveries were significantly different between cases and controls (Table 3). Among women who had a vaginal delivery in a subsequent pregnancy, OASIS in the first delivery significantly increased the tear recurrences. Tears recurred in 6.6% of cases vs. 1.7% of controls (*p* < 0.001) in Norway; in 4.5% of cases vs. 0.7% of controls (*p* = 0.001) in Sweden; and in 2.1% of cases vs. 0.8% of controls (*p* < 0.05) in Finland (Table 3). At the time of data collection, OASIS were more frequently associated with operative deliveries, than among those with uncomplicated spontaneous vaginal deliveries [8]. Further, the total incidence of OASIS in Sweden was seven times higher than in Finland, but for low-risk delivery the risk was 13-times higher in Sweden compared to Finland [8].

**Table 2:** The births in subsequent pregnancy among women with (cases) and without (controls) obstetric anal sphincter tear in the first pregnancy

	Finland		Norway		Sweden	
	Cases	Controls	Cases	Controls	Cases	Controls
*N*	1001	1001	10,327	10,327	15,270	5270
No births	346	347	3732	3686	4650	4740
At least one birth	655	654	6595	6641	10,620	10,530
	65.4%	65.3%	63.9%	64.3%	69.5%	69.0%
	*p* = 0.963		*p* = 0.505		*p* = 0.264

**Table 3:** The obstetric anal sphincter tear frequency and rate of cesarean delivery in a subsequent delivery among women with (cases) or without (controls) obstetric anal sphincter tears in the first delivery

Country	Group	OASIS (%)	Cesarean delivery (%)	Planned cesarean delivery (%)
Finland	Cases	14/655 (2.1)	334/655 (50.2)	308/655 (47.0)
	Controls	5/654 (0.8)	93/654 (14.2)	40/654 (6.1)
	*p*	0.038	< 0.001	< 0.001
Norway	Cases	433/6595 (6.6)	1080/6595 (16.4)	758/6595 (11.5)
	Controls	111/6641 (1.7)	527/6641 (7.9)	222/6641 (3.3)
	*p*	< 0.001	< 0.001	< 0.001
Sweden	Cases	480/10,620 (4.5)	1733/10,620 (16.3)	1394/10,620 (13.1)
	Controls	76/10,530 (0.7)	631/10,530 (6.0)	353/10,530 (3.4)
	*p*	< 0.001	< 0.001	< 0.001

*OASIS* obstetric anal sphincter injuries

There were no statistically significant differences between cases and controls in the median number of deliveries during the follow-up period; i.e., one in cases and controls in Finland, two in cases and controls in Sweden, and one in cases and controls in Norway. There were no significant differences between cases and controls in the median years to the next delivery, in Finland (1.9 vs. 1.9 years, respectively), in Sweden (3.0 vs. 3.0 years, respectively), or in Norway (3.4 vs. 3.3 years, respectively).

The number of deliveries in Sweden was significantly higher among controls than among cases; more than two children were delivered in 4.2% of cases and 5.1% of controls (*p* = 0.002). The same was true even in Norway (Table 4). However, no significant difference was observed between cases and controls in Finland (Table 4).

**Table 4:** The number of deliveries compared with cases (previous obstetric anal sphincter injury) and controls (no obstetric anal sphincter injury), during follow-up (%)

Deliveries	Finland			Norway			Sweden		
	Cases	Controls	*p*	Cases	Controls	*p*	Cases	Controls	*p*
1	424 (64.7)	429 (656)		4704 (71.3)	4623 (69.6)		7693 (72.4)	7414 (70.4)	
2	174 (26.6)	162 (24.8)		1575 (23.9)	1608 (24.2)		2476 (23.3)	2574 (24.4)	
More than 2	87 (8.7)	63 (9.6)	0.685	36 (.8)	410 (6.2)	0.001	451 (4.2)	542 (5.1)	< 0.001
Total	655	654		6595	6641		10,620	10,530	

In Finland, it was possible to use the data from induced abortions and sterilizations. We found that women tended to divorce more often among the study group than controls with grade 4 tears in Finland (*p* = 0.08). However, there was no difference in adoption rate between cases and controls.

## Discussion

We have shown that there is a threefold increase of repeated OASIS in the low-risk, and four- to sixfold increases in the high-risk population. This means that 2.1% in the low-risk and 4.5–6.6% in the high-risk population will suffer from OASIS after a sphincter injury during their first delivery. The OASIS rate in a subsequent delivery after earlier OASIS in a high-risk population compared to a low-risk population (two- to threefold increase) is a little bit less than the overall difference in OASIS rate (fourfold increase). The reason for this could be the low OASIS rate in Finland compared to the two other Scandinavian countries. Because, if an OASIS takes place during the first birth and also during the subsequent birth in Finland, it is probable that the event of a rupture is independent of the midwives' and doctors' supporting techniques.

We found that the cesarean delivery rate for a subsequent pregnancy was significantly higher after an OASIS than among controls, in all three countries. This finding was consistent with earlier reports [13]. Interestingly, the cesarean rate was highest in Finland, which had a low incidence of OASIS. The elective cesarean rate did not differ substantially in women without a serious tear in all three countries, but acute cesareans were more common in Norway and Sweden for cases. To find the optimal cesarean rate for the prevention of recurrent OASIS in the Nordic countries there is need for more scientific research. Our results confirmed earlier findings that women who underwent a cesarean delivery had an increased likehood of OASIS compared to women that had not undergone a cesarean delivery [14, 15].

. The prevalence of new tears varied between 2.1% in Finland to 6.6% in Norway. Thus, we found a four- or fivefold increased likehood of a subsequent OASIS in Norway and Sweden, in contrast, the likelihood was threefold higher in Finland. These results were consistent with those published previously [16, 17]. However, one previous study reported no increase in the OASIS rate after OASIS in the first pregnancy [18]. The prevalence of OASIS in a new pregnancy among controls was low both in Finland (0.8%) and Sweden (0.7%) but significantly higher in Norway (1.8%).

We have previously showed that the OASIS rate was largely different among different Nordic countries [8, 19]. This difference may be due to the difference in manual support techniques. It is also possible that the use of a correct episiotomy (lateral or mediolateral) in instrumental deliveries played an important role [20–24]. Until 2005, Norway had the highest OASIS rate in the Nordic countries. The risk of OASIS increased considerably in Norway from 1967 to 2004. Changes in risk factors studied could only partially explain the increase [25]. A national strategy to reduce the rate of OASIS was initiated in 2005 by the Norwegian Board of Health. Successful intervention programs [26, 27] have improved OASIS statistics in Norway, and currently, the national mean prevalence is 1.8% (MBR data for 2018). Similar change can be seen in Sweden, and the OASIS rate today is 2.5% (data from National Board of Health and Welfare 2018).

We found that the number of children born to women who have had OASIS was unchanged after one or two subsequent deliveries but was significantly lower than the number born to matched controls after more than two subsequent deliveries in the high-risk populations in Norway and Sweden. This finding has not been reported previously, perhaps due to the short follow-up periods and small cohorts of previous studies. However, these results must be confirmed in future studies with a larger cohort.

The major strength of this study was the large sample size. We included deliveries from all maternity and delivery units in three Nordic countries. Furthermore, these databases have been used in earlier studies [27], and the OASIS rates were shown to be highly valid, which could justify future large-scale epidemiologic studies based on these databases, both in Finland and Norway [28]. However, this study was limited by the typical problems associated with registry-based studies where the data is dependent on many incidents and registration levels, and one mistake in each step could result in wrongful information. In addition, it was not possible to obtain data on the different grades of OASIS from birth registries in Norway and Sweden. For future research, the statistical multivariate analysis would have been useful in aim to decrease the impact of these confounding factors on the results.

There is a need to improve the prevention, diagnostics, and treatment [5] of OASIS, and thus, improve the obstetric consequences for women after delivering a child. An OASIS during the first delivery increases the risk of incontinence symptoms, even after the second delivery [29, 30]. Furthermore, it has been estimated that between 17 and 24% of women developed worsening fecal symptoms after a second vaginal delivery, following a previous OASIS [32]. When a second OASIS occurs, it is much more challenging to repair with good functional results. Therefore, once again, it is easy to agree with Samarasekera et al. (2008) that the important thing is to prevent the first tear.
